# Rosmarinic Acid Induces Vasorelaxation via Endothelium-Dependent, Potassium Channel-Related, and Calcium-Modulated Pathways: Evidence from Rat Aortic Rings

**DOI:** 10.3390/biomedicines13122936

**Published:** 2025-11-29

**Authors:** Serdar Sahinturk, Naciye Isbil

**Affiliations:** Department of Physiology, Faculty of Medicine, Bursa Uludağ University, 16059 Bursa, Turkey; nisbil@uludag.edu.tr

**Keywords:** organ bath, nitric oxide, rosmarinic acid, thoracic aorta, vasorelaxation

## Abstract

**Background:** Hypertension and its complications are a major global health problem, and natural compounds with vasorelaxant effects are being investigated as potential antihypertensive agents. **Objective:** This study aimed to determine whether rosmarinic acid (RA) induces vasorelaxation in the rat thoracic aorta and to elucidate the underlying mechanisms. **Methods:** Isolated thoracic aortic rings, with or without endothelium, were precontracted with phenylephrine and subsequently exposed to cumulative concentrations of RA. The roles of endothelium-derived factors, potassium channels, and calcium signaling were evaluated using selective pharmacological inhibitors and activators. In addition, the involvement of the AMPK pathway, adenylate cyclase/cAMP pathway, PKC signaling, β-adrenergic receptors, muscarinic receptors, and angiotensin II in RA-induced vasorelaxation was investigated. **Results:** RA induced a concentration-dependent vasorelaxation in endothelium-intact thoracic aortic rings (*p* < 0.001; pD_2_ = 7.67 ± 0.04). The vasorelaxant effect of RA was attenuated in endothelium-denuded vessels (pD2: 5.26 ± 0.18). The relaxation response was significantly attenuated by inhibitors of the PI3K/Akt/eNOS/NO/cGMP pathway and by blockers of BK_Ca_, IK_Ca_, and Kv potassium channels (*p* < 0.001). Furthermore, RA markedly inhibited both extracellular Ca^2+^ influx and intracellular Ca^2+^ release from the sarcoplasmic reticulum (*p* < 0.001). RA incubation also significantly reduced the contractions induced by angiotensin II (Ang II) and by the PKC activator PMA (*p* < 0.001). Other tested pathways had no significant influence on the vasorelaxant effect of RA (*p* > 0.05). **Conclusions:** These findings demonstrate that rosmarinic acid induces both endothelium-dependent and endothelium-independent vasorelaxation in the rat thoracic aorta through activation of the PI3K/Akt/eNOS/NO/cGMP pathway, opening of BK_Ca_, IK_Ca_, and Kv potassium channels, and suppression of Ca^2+^ mobilization. Additionally, inhibition of PKC- and angiotensin II-mediated vascular contraction contributes to RA-induced vasorelaxation. RA may therefore have therapeutic potential in the management of hypertension.

## 1. Introduction

Cardiovascular diseases are among the leading causes of morbidity and mortality worldwide, with hypertension-related complications, including stroke, heart failure, and coronary artery disease, being the most prevalent and deadly [1,2,3]. Affecting one in three adults globally and responsible for one in five deaths, hypertension remains a major public health problem. Despite the availability of various antihypertensive drugs, only about half of patients achieve adequate blood pressure control, and many experience serious adverse effects, including cancer, diabetes, and kidney failure [1,2,3,4,5,6,7,8,9,10]. Therefore, there is an urgent need for the development of novel therapeutic agents with higher efficacy and improved safety profiles.

Vascular smooth muscle relaxation is regulated by multiple mechanisms, including signals from endothelial cells—such as nitric oxide (NO) and prostacyclin (PGI_2_)—as well as receptor-mediated pathways. The phosphatidylinositol-3-kinase (PI3K)/protein kinase B (Akt) signaling axis plays a well-established role in endothelium-dependent aortic relaxation by activating eNOS and stimulating NO production [11]. NO diffuses into smooth muscle cells, activates soluble guanylate cyclase (sGC), increases cyclic guanosine monophosphate (cGMP) production, and, through protein kinase G (PKG), inhibits Ca^2+^ entry, thereby promoting relaxation [12]. Prostacyclin activates adenylate cyclase, increasing cAMP levels and stimulating protein kinase A (PKA), thereby contributing to vasorelaxation [13]. Activation of β-adrenergic receptors similarly stimulates cAMP production in smooth muscle cells, leading to relaxation, while muscarinic receptor activation on the endothelium can enhance NO-mediated responses [14]. Reducing intracellular Ca^2+^ levels—through inhibition of voltage-dependent calcium channels, reuptake into the sarcoplasmic reticulum, or extrusion from the cell—also supports relaxation [15]. Activation of potassium channels hyperpolarizes the membrane, decreasing Ca^2+^ influx [16]. In contrast, angiotensin II, a major vasoconstrictor, activates G-protein-coupled AT_1_ receptors, stimulating phospholipase C (PLC)/Inositol 1,4,5-trisphosphate (IP_3_) pathways, which increase Ca^2+^ sensitivity and influx, promoting smooth muscle contraction [17].

*Rosmarinus officinalis* Linn. (rosemary), a member of the Lamiaceae family, has long been used in traditional medicine for the treatment of ailments such as stomachache, dysmenorrhea, headache, spasms, rheumatic pain, nervous agitation, epilepsy, and depression. This plant contains numerous bioactive compounds, including volatile oils, alkaloids, terpenoids, and flavonoids. Phytochemical analyses have identified phenolic diterpenes, phenolic acids, and triterpenes—such as carnosic acid, rosmarinic acid (RA), carnosol, rosmanol, betulinic acid, and ursolic acid—as the principal active constituents [18,19,20,21,22,23]. Among these, RA is particularly notable for its broad pharmacological activities, including antioxidant, antiviral, antimetabolic, anticancer, antimutagenic, neuroprotective, and antinociceptive effects [22,23,24].

The diverse biological effects of RA have been well-documented. However, whether RA exerts a relaxant effect on vascular smooth muscle and the underlying mechanisms remain unknown. Therefore, this study aims to elucidate the effects of RA on vascular smooth muscle contractility and to investigate the underlying mechanisms using isolated rat thoracic aorta preparations and the organ bath technique. The study further explores the potential involvement of several pathways, including potassium and calcium channels, prostacyclin production, β-adrenergic and muscarinic receptor activation, adenylate cyclase–cAMP pathway activation, inhibition of protein kinase C (PKC) and the renin–angiotensin system, as well as the PI3K/Akt/eNOS/NO/cGMP signaling cascade.

## 2. Materials and Methods

### 2.1. Ethical Approval and Experimental Animals

Ethics committee approval for the study was obtained from the Bursa Uludağ University Animal Experimentation Local Ethics Committee (No: 2024-18/04). Fifty 10- to 12-week-old male Sprague Dawley rats, obtained from the Bursa Uludağ University Experimental Animal Breeding Application and Research Center, were used as experimental animals. The rats were cared for until the experiment day, observing animal welfare, and in accordance with national and international guidelines. The experiments were conducted in the Cardiovascular Research Laboratory in the Department of Physiology, Department of Basic Medical Sciences, Faculty of Medicine, Bursa Uludağ University. The number of rats used in the study was 50. The rats were randomly assigned to experimental groups to ensure an equal number of animals per group. Four vascular rings were obtained from each rat. To obtain *n* = 6 or *n* = 8 vascular rings per group, two rats were used for each experimental group. After confirming tissue viability and endothelial integrity, the rings were washed three times to remove any residual drugs, and experimental protocols were conducted following a 1-h equilibration period. Vascular rings treated with inhibitors were not reused.

### 2.2. General Study Design and Experimental Protocol

This study used an isolated organ bath system with four 10-mL chambers (four channels) (MAY IOBS99 isolated organ bath, Commat Ltd., Ankara, Turkey/Biopac MP36 data acquisition and analysis system, BIOPAC Systems, Inc., Goleta, CA, USA). The thoracoabdominal regions of rats anesthetized with ketamine/xylazine were opened, and their thoracic aortas were rapidly removed. Thoracic aortic tissues were placed in Petri dishes containing freshly prepared Krebs solution. 3–4 mm long vascular rings were prepared from the vessels, carefully cleared of perivascular tissue. The vascular rings were placed in glass chambers containing Krebs solution (37 °C, pH: 7.4) gassed with a mixture of 95% O_2_ and 5% CO_2_ in the isolated organ bath system using surgical suture and vascular hanging apparatus. The resting tension was set at 1 g. A 60-min equilibration period was applied for the tissues to adapt to the environment. During this period, the Krebs solution in the chambers was renewed every 10 min. After the equilibration period, the viability of the vascular rings and the integrity of the endothelium were checked. A relaxation response induced by 1 µM acetylcholine (ACh) in vessels constricted with 1 µM phenylephrine (PE) exceeding 60% was considered to indicate that the endothelium was intact. In some groups, the vascular endothelium was gently removed by rubbing with a toothpick. The vascular smooth muscle relaxation achieved by ACh in these groups was below 10%, indicating successful endothelium removal. Subsequently, active and inhibitory substances were administered to separate vascular rings for each group. Drug administration was performed precisely in Krebs solution using an adjustable automatic pipette. Tension changes resulting from isometric contraction and relaxation responses in the vascular rings were recorded. Percentages of the tension values obtained were calculated by assuming a plateau-phase tension obtained with PE, which was used to achieve precontraction, as 100%. All experimental protocols were implemented in accordance with previous studies [25,26,27,28,29,30,31,32,33,34,35].

### 2.3. Investigation of the Roles of Endothelium-Dependent Pathways, Other Possible Mechanisms, and Potassium Channels in RA-Induced Vasorelaxation

All isolated organ bath experiments were performed as described in previous studies. Following the equilibration period, after a stable tension was established in the PE (1 µM)-contracted vessels, RA was applied cumulatively (10^−10^–10^−5^ M). To determine the mechanisms of action (PI3K/Akt/eNOS/NO/cGMP pathway, cyclooxygenase (COX) pathway, potassium channels, adenylate cyclase–cAMP pathway, muscarinic receptors, and beta-adrenergic receptors, among others), inhibitor and blocker applications were performed 30 min before PE application. Drug concentrations are given in Table 1. Concentration-response curves were obtained, and at this stage, the pD_2_ and Rmax (pD_2_: efficient concentration, Rmax: maximal relaxation) values were calculated. pD_2_ is a measure of a drug’s potency and is calculated as the negative logarithm of the concentration (EC_50_) that produces 50% of the drug’s maximal effect. A lower EC_50_, meaning the drug is effective at a lower concentration, corresponds to a higher pD_2_, indicating greater potency. Experimentally, pD_2_ is determined by measuring the drug’s effect on tissue at different concentrations, plotting a concentration-response curve, identifying the EC_50_, and then taking its negative logarithm.

### 2.4. Investigating the Role of Extracellular Calcium Sources in RA-Induced Vasorelaxation

To determine the involvement of extracellular Ca^2+^ influx and calcium channels in RA-induced vasorelaxation, thoracic aortic rings were incubated with 10 µM RA in Ca^2+^-free Krebs solution. Tissues were then exposed to 60 mM KCl, and concentration-response curves were generated against cumulative additions of calcium chloride (10^−6^–10^−2^ M). In another set of experiments, tissues were incubated with 10 µM RA for 45 min before the addition of the voltage-gated calcium channel opener Bay K8644 (a L-type calcium channel activator) (10 µM) in calcium-containing Krebs solution. The thoracic aortic rings were preincubated with RA (10 µM) for 45 min, and then 10 µM ionomycin (a calcium ionophore; it opens membrane pores that facilitate calcium influx) was added to confirm the effect of RA on ionomycin-stimulated vascular contraction. To rule out whether ionomycin-induced vascular contraction involves L-type calcium channels, the same experimental procedure was repeated in the presence of 10 µM nifedipine.

### 2.5. Investigating the Role of Intracellular Calcium Sources in RA-Induced Vasorelaxation

The effect of RA on the release of intracellular calcium stores was determined by pre-incubating tissues with RA (10 µM) for 45 min and then adding 20 mM caffeine (ryanodine receptor activator) or 10 µM cyclopiazonic acid (CPA) (a specific Ca^2+^-ATPase inhibitor) to calcium-containing Krebs solution.

### 2.6. Investigating the Role of the PKC Signaling Pathway in RA-Induced Vasorelaxation

The thoracic aortic rings were preincubated with RA (10 µM) for 45 min, and then phorbol 12-myristate 13-acetate (PMA) (100 µM), a PKC activator, was added to confirm the effect of RA on PKC-stimulated vascular contraction.

### 2.7. Investigation of the Effect of RA on Ang II-Induced Vasorelaxation

Thoracic aortic tissues were incubated with RA (10 µM) for 20 min. Ang II (10^−9^–10^−6^ M) was then applied cumulatively.

### 2.8. Drugs

Drugs and chemicals were bought from different businesses. Sigma-Aldrich was the supplier of PE, indomethacin, tetraethylammonium, ketamine, and barium chloride (BaCl_2_). BLDpharm (BLD Pharmatech Ltd., Shanghai, China) was the supplier of RA, NG-Nitroarginine methyl ester hydrochloride (L-NAME), PMA, 1H-[1,2,4]oxadiazolo-[4, 3-a]quinoxalin-1-one (ODQ), LY294002, ACh, dorsomorphin, glyburide, and methylene blue. GlpBio (GlpBio Technology Inc., Montclair, NJ, USA) was the supplier of anandamide, iberiotoxin, Ang II, ionomycin, propranolol, XE-991, TRAM-34, xylazine, and tricirbine. We bought 4-aminopyridine (4-AP), atropine, and nifedipine from Macklin (Shanghai Macklin Biochemical Co., Ltd., Shanghai, China). We bought caffeine from Kimyalab (BeyanLab Ltd., İstanbul, Turkey). Apollo Scientific (Apollo Scientific Ltd., Manchester, UK) sold CPA. Aaron Chemicals (Aaron Chemicals LLC, San Diego, CA, USA) was the seller of Bay K8644 and SQ22536. Cayman Chemical (Cayman Chemical, Ann Arbor, MI, USA) was the supplier of KT5823. We bought Apamin from MedChem Express (MedChemExpress LLC, Monmouth Junction, NJ, USA).

PE (1 μM), ACh (1 μM), TEA (1 mM), methylene blue (10 μM), L-NAME (100 μM), BaCl_2_ (10 μM), TRAM-34 (1 μM), iberiotoxin (10 nM), 4-AP (1 mM), apamin (100 nM), caffeine (20 mM), nifedipine (1 μM), LY294002 (10 μM), XE-991 (10 μM), KT5823 (1 μM), and SQ22536 (50 μM) were dissolved in distilled water. RA (10^−10^–10^−5^ M), tricirbine (10 μM), dorsomorphin (1 μM), glyburide (10 μM), ODQ (10 μM), anandamide (10 μM), indomethacin (10 μM), PMA (100 μM), Ang II (10^−9^–10^−6^ M), nifedipine (10 μM), atropine (1 μM), propranolol (1 μM), Bay K8644 (10 μM), and CPA (10 μM) were all dissolved in DMSO. DMSO had no effect on the contraction or relaxation of VSM, and its final concentration in the Krebs solution was less than 0.1%. All medication dosages were established by earlier research. Drug concentrations and references are presented in Table 1.

### 2.9. Statistical Analyses

Before the study began, a power analysis was performed, and the sample size was determined. Cohen’s d was used to calculate the difference between groups, yielding an effect size of 0.30. With a power of 0.80 and α = 0.05, the minimum sample size was estimated at n = 5 per group. Power analysis was performed using GPower 3.1. The obtained data were statistically analyzed using GraphPad Prism (version 8.0). Data were expressed as mean ± standard deviation (SD). The variable “*n*” represented the total number of thoracic aortic rings used in all study groups. RA-mediated relaxation responses were expressed as a percentage of the maximum plateau phase contraction induced by PE. Student’s two-tailed, paired *t*-test was used for statistical comparisons between two groups. One-way ANOVA followed by a Dunnett’s post hoc test was used for statistical comparisons of multiple groups. Two-way ANOVA was used to compare concentration-response curves. The Bonferroni test was selected as a post hoc test.

## 3. Results

### 3.1. RA-Induced Vasorelaxation in Rat Thoracic Aorta

**Figure 1:**
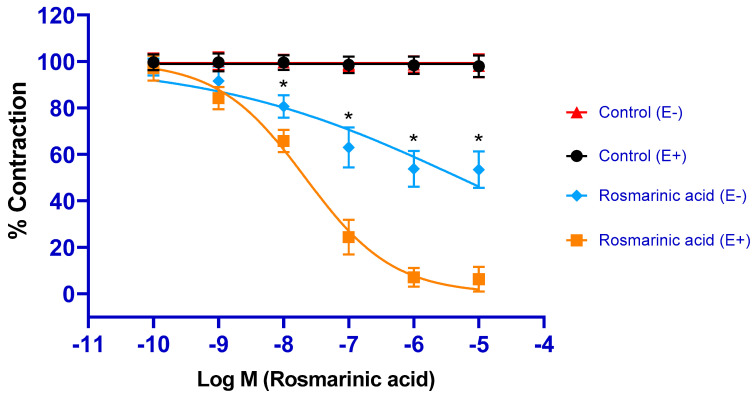
Log concentration-response curves for the vasorelaxant effects of rosmarinic acid (10^−10^–10^−5^ M) in the absence or presence of endothelium. Endothelium-intact (E+) rings precontracted with PE (1 µM) relaxed in response to rosmarinic acid (10^−10^–10^−5^ M). In endothelium-denuded (E) rings, rosmarinic acid-induced relaxation was significantly, but not completely, diminished. Data shown are the mean ± SD (*n* = 8). * *p* < 0.001 vs. the control group. (2-way ANOVA followed by a Bonferroni post hoc test).

RA administration caused concentration-dependent vasorelaxation in PE-precontracted endothelium-intact aortic rings (pD_2_: 7.67 ± 0.04). In endothelial-removed aortic rings, the vasorelaxant effect of RA was largely reduced. However, the vasorelaxant effect was not completely eliminated (pD_2_: 5.26 ± 0.18) (Figure 1).

### 3.2. Roles of Endothelium-Dependent Mechanisms in RA-Induced Vasorelaxation

**Figure 2:**
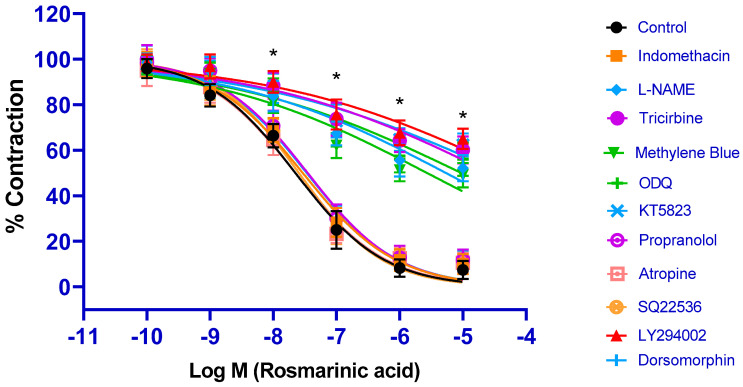
Log concentration–response curves for the vasorelaxant effects of rosmarinic acid (10^−10^–10^−5^ M) in the absence or presence of indomethacin (10 µM), L-NAME (100 µM), tricirbine (10 μM), methylene blue (10 μM), ODQ (10 μM), KT5823 (1 μM), propranolol (1 μM), atropine (1 μM), SQ22536 (50 μM), LY294002 (10 μM), and dorsomorphine (1 μM) in endothelium-intact aortic rings in comparison to control. Data shown are the mean ± SD (*n* = 8). * *p* < 0.001 vs. the control group. (2-way ANOVA followed by a Bonferroni post hoc test).

All inhibitors related to the PI3K/Akt/eNOS/NO/sGC/cGMP/PKG signaling pathway (PI3K inhibitor LY294002, Akt inhibitor tricirbine, eNOS inhibitor L-NAME, sGC inhibitor ODQ, cGMP inhibitor methylene blue, and PKG inhibitor KT5823) significantly inhibited RA-induced vasorelaxation (*p* < 0.001). pD_2_ values were found as 4.17 ± 0.11, 4.57 ± 0.18, 5.27 ± 0.14, 5.02 ± 0.09, 5.57 ± 0.12, and 4.37± 0.11, respectively. Application of the COX 1/2 inhibitor indomethacin did not produce any significant inhibition (*p* > 0.05) (pD_2_: 7.53 ± 0.05) (Figure 2).

### 3.3. Contributions of Other Potential Signaling Pathways to RA-Induced Vasorelaxation

Incubations with the AMPK signaling pathway inhibitor dorsomorphin, the beta-adrenergic receptor blocker propranolol, the muscarinic receptor blocker atropine, and the adenylate cyclase/cyclic adenosine monophosphate inhibitor SQ22536 did not result in a significant change in the vasorelaxant effect of RA (*p* > 0.05). pD_2_ values were found to be 7.47 ± 0.16, 7.46 ± 0.05, 7.65 ± 0.09, and 7.59 ± 0.08, respectively (Figure 2).

### 3.4. Contribution of Potassium Channel Activation to RA-Induced Vasorelaxation

**Figure 3:**
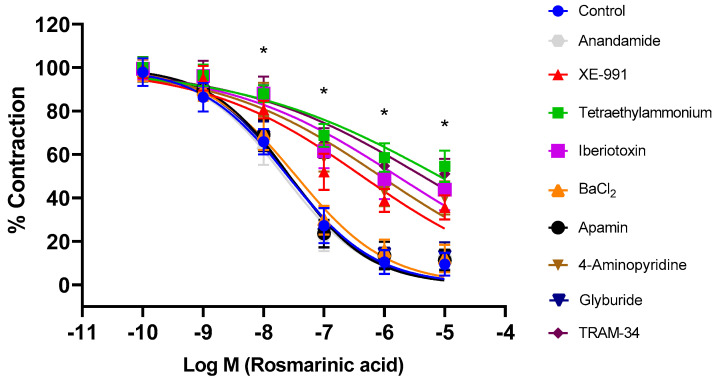
Log concentration–response curves for the vasorelaxant effects of rosmarinic acid (10^−10^–10^−5^ M) in the absence or presence of tetraethylammonium (nonselective K^+^ channel blocker; 1 mM), iberiotoxin (BK_Ca_ channel blocker; 10 nM), TRAM-34 (IK_Ca_ channel blocker; 1 µM), 4-aminopyridine (Kv channel blocker; 1 mM), XE-991 (Kv7.1–7.5 channel blocker; 1 µM), glyburide (K_ATP_ channel blocker; 10 µM), apamin (SK_Ca_ channel blocker; 100 nM), anandamide (K_2P_ channel blocker; 10 µM), and BaCl_2_ (Kir channel blocker; 10 µM) in endothelium-intact aortic rings in comparison to control. Data shown are the mean ± SD (*n* = 8). * *p* < 0.001 vs. the control group. (2-way ANOVA followed by a Bonferroni post hoc test).

**Figure 4:**
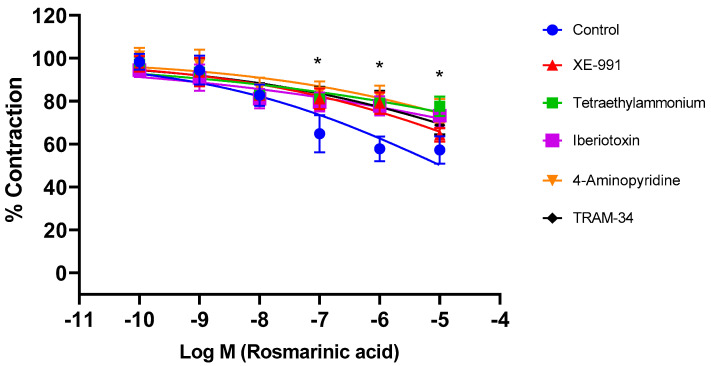
Log concentration–response curves for the vasorelaxant effects of rosmarinic acid (10^−10^–10^−5^ M) in the absence or presence of tetraethylammonium (nonselective K^+^ channel blocker; 1 mM), iberiotoxin (BK_Ca_ channel blocker; 10 nM), TRAM-34 (IK_Ca_ channel blocker; 1 µM), 4-aminopyridine (Kv channel blocker; 1 mM), and XE-991 (Kv7.1–7.5 channel blocker; 1 µM) in endothelium-denuded aortic rings in comparison to control. Data shown are the mean ± SD (*n* = 6). * *p* < 0.001 vs. the control group. (2-way ANOVA followed by a Bonferroni post hoc test).

Aortic rings were incubated with selective and nonselective potassium channel blockers for 30 min. Afterwards, RA was applied cumulatively to the vessels constricted with PE. The nonselective potassium channel inhibitor tetraethylammonium, the large-conductance calcium-activated potassium channel (BK_Ca_) blocker iberiotoxin, the intermediate-conductance calcium-activated potassium channel (IK_Ca_) blocker TRAM-34, the voltage-gated potassium channel (Kv) blocker 4-AP, and the KV7.1–7.5 potassium channel blocker XE-991 significantly inhibited RA-induced vasorelaxation (*p* < 0.001). pD_2_ values in these groups were determined as 5.09 ± 0.15, 5.79 ± 0.12, 5.37 ± 0.14, 6.07 ± 0.11, and 6.37 ± 0.10, respectively. In contrast, the small-conductance calcium-activated potassium channel (SK_Ca_) blocker apamin, the ATP-sensitive potassium channel (K_ATP_) blocker glyburide, the membrane 2-pore potassium channel (K_2p_) blocker anandamide, and the inward-rectifying potassium channel (Kir) blocker BaCl_2_ did not produce a significant effect (*p* > 0.05). pD2 values in these groups were found to be, 7.57 ± 0.05, 7.59 ± 0.06, 7.66 ± 0.06, and 7.46 ± 0.07, respectively (Figure 3). To demonstrate that potassium channels play a direct role in RA–induced vasorelaxation, experiments were performed in endothelium-denuded vessels. Treatment with TEA (pD_2_: 4.98 ± 0.22), iberiotoxin (pD_2_: 1.67 ± 0.12), TRAM-34 (pD_2_: 2.96 ± 0.17), 4-AP (pD_2_: 2.43 ± 0.18), and XE-991 (pD_2_: 3.52 ± 0.19) significantly reduced the vasorelaxant effect of RA (*p* < 0.001) (Figure 4).

### 3.5. Role of Extracellular Calcium Sources

**Figure 5:**
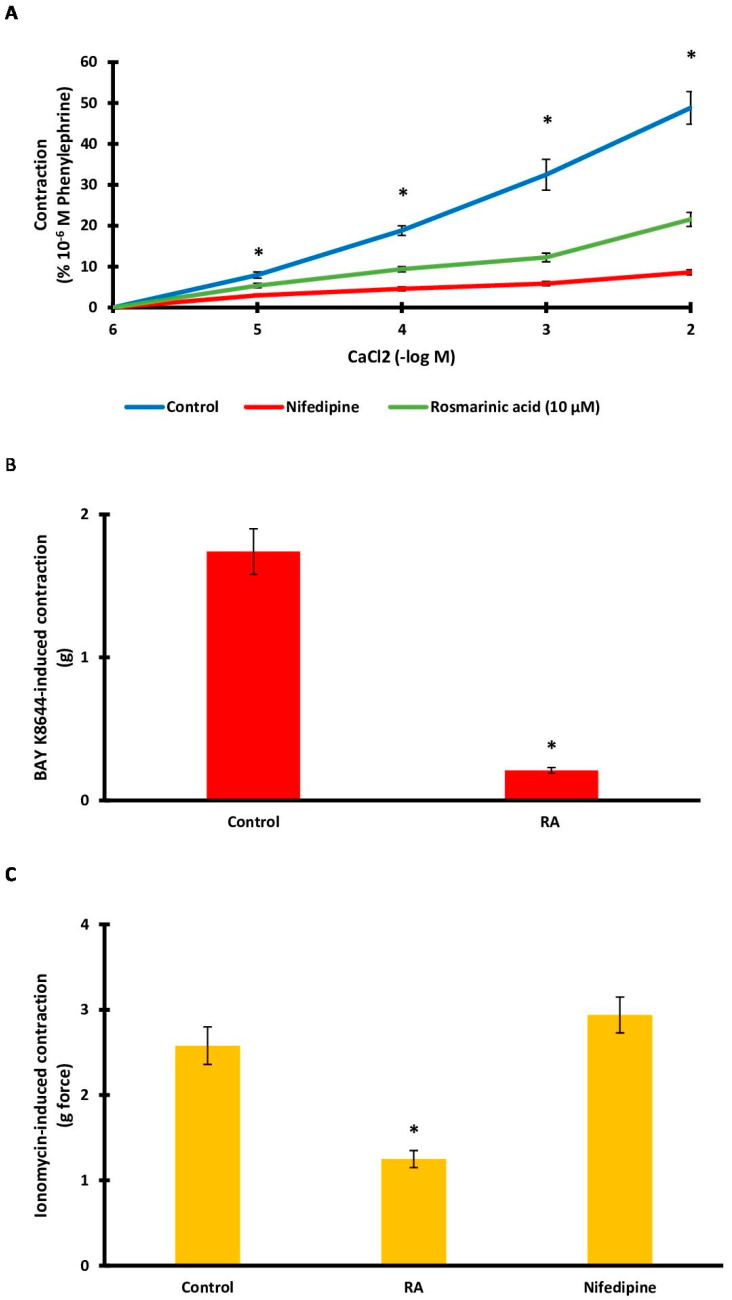
(**A**). Log concentration–response curves for CaCl_2_ (10^−6^–10^−2^ M)-induced contractions in the presence of rosmarinic acid (RA; 10 μM) in comparison with control. Data shown are the mean ± SD (*n* = 6). * *p* < 0.001 vs. the control group. (2-way ANOVA followed by a Bonferroni post hoc test). (**B**). Effect of RA (10 μM) on Ca^2+^ influx via L-type Ca^2+^ channel. A contraction response induced by Bay K8644 (10 µM) was obtained. RA (10 μM) was applied to the rings before inducing contractions using Bay K8644 (10 µM). Data shown are the mean ± SEM (*n* = 6). * *p* < 0.001 vs. the control group. (Student’s 2-tailed, paired *t*-test). (**C**). Bar charts show contractions induced by ionomycin (10 μM) in aortic rings in the absence and presence of RA (1 μM). Data shown are the mean ± SEM (*n* = 6). * *p* < 0.001 vs. the control group. (One-way ANOVA followed by a Dunnett’s post hoc test).

Aortic rings contracted upon exposure to CaCl_2_ (10^−6^–10^−2^ M). The level of contraction (48.82 ± 3.98%) decreased significantly after RA incubation (21.55 ± 1.72%) (*p* < 0.001). Nifedipine, used as a positive control, also caused a large decrease in tension values (8.60 ± 0.63%) (Figure 5A). The L-type calcium channel agonist Bay K8644 was administered to determine the role of L-type calcium channels. The level of contraction (1.74 ± 0.16 g) decreased significantly following RA incubation (0.21 ± 0.02 g) (*p* < 0.001) (Figure 5B). Ionomycin was used to demonstrate the effect of calcium influx through membrane pores in the cell membrane. Similarly, RA incubation significantly reduced (1.25 ± 0.10 g) ionomycin-induced contraction (2.58 ± 0.22 g) (*p* < 0.001). However, nifedipine administration did not significantly affect the level of ionomycin-induced contraction (2.94 ± 0.21 g) (*p* > 0.05) (Figure 5C).

### 3.6. Role of Intracellular Calcium Sources

**Figure 6:**
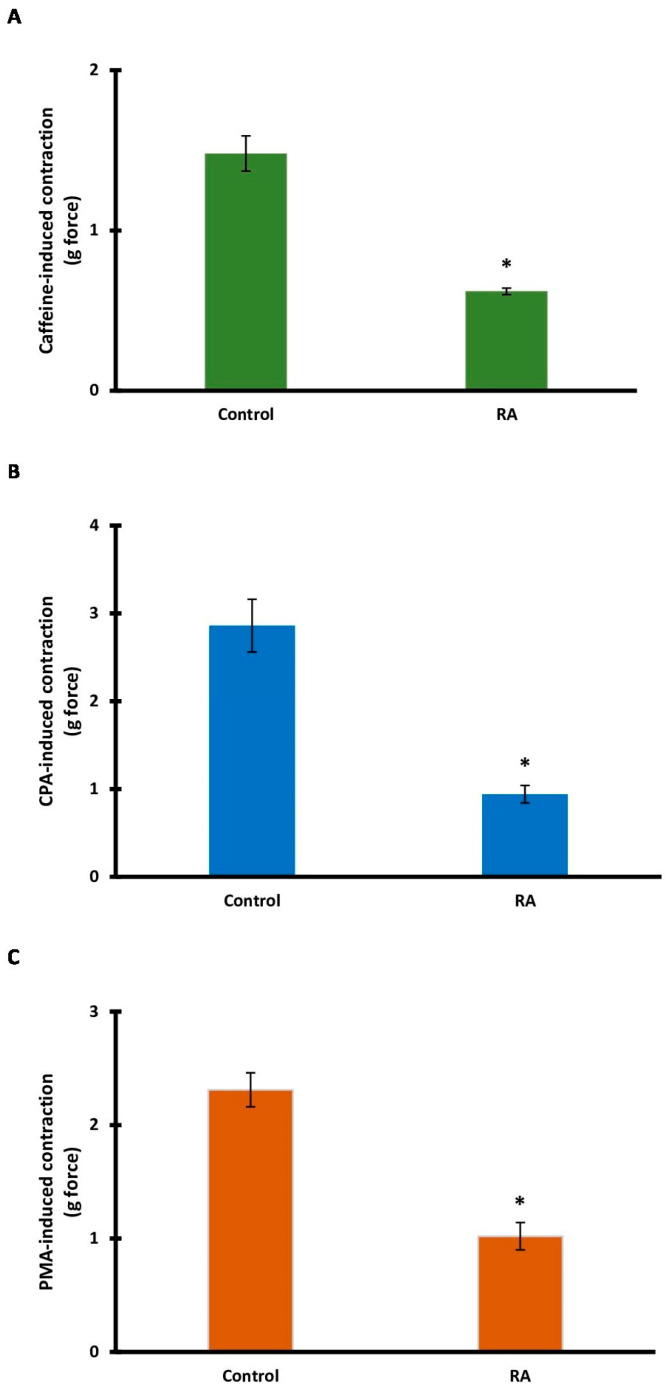
The contributions of intracellular calcium sources and protein kinase C in rosmarinic acid (RA)-induced vasorelaxation. Bar charts show contractions induced by caffeine (20 mM) (**A**), cyclopiazonic acid (CPA; 10 μM) (**B**), and phorbol 12-myristate 13-acetate (PMA; 100 μM) (**C**) in aortic rings in the absence and presence of RA (10 μM). Data shown are the mean ± SD (*n* = 6). * *p* < 0.001 vs. the control group. (Student’s 2-tailed, paired *t*-test).

To determine the role of intracellular calcium sources, the effect of RA incubation on caffeine- and CPA-induced contractions was evaluated. RA incubation significantly inhibited (0.32 ± 0.04 g) caffeine-induced contractions (1.26 ± 0.09 g) (*p* < 0.001) (Figure 6A). CPA-induced contractions (3.12 ± 0.17 g) were also significantly reduced (0.63 ± 0.08 g) by RA incubation (*p* < 0.001) (Figure 6B).

### 3.7. Role of the PKC Signaling Pathway

PMA, an activator of the PKC signaling pathway, was used. After RA incubation, PMA-induced tension decreased significantly (*p* < 0.001). The mean tension in the PMA (control) group was 2.31 ± 0.15 g, while the mean tension in the RA group was 1.02 ± 0.12 g (Figure 6C).

### 3.8. Role of the Renin-Angiotensin System

**Figure 7:**
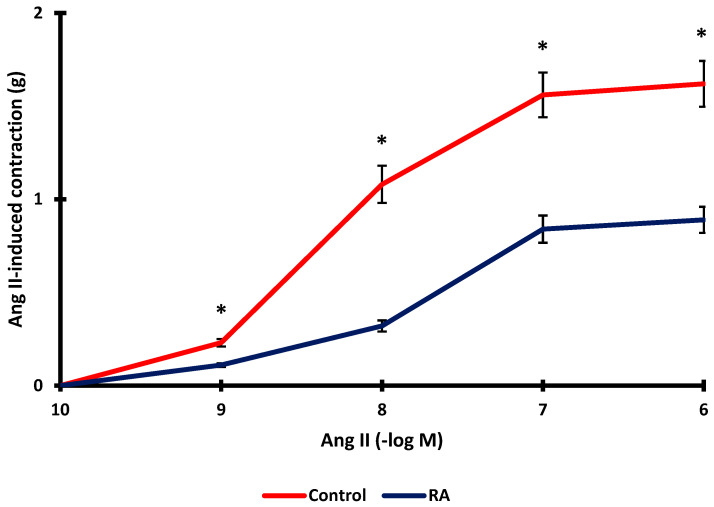
Effect of rosmarinic acid (RA; 10 µM) on aortic rings constricted by Ang II (10^−9^–10^−6^ M). Data shown are the mean ± SD (*n* = 6). * *p* < 0.001 vs. the control group. (2-way ANOVA followed by a Bonferroni post hoc test).

It was investigated whether RA incubation inhibited Ang II-induced vascular contraction. Ang II-induced vascular contraction decreased significantly after RA incubation (*p* < 0.001). While the maximum contraction level obtained with Ang II was 1.62 ± 0.12 g, the maximum contraction level after RA incubation was determined to be 0.89 ± 0.07 g (Figure 7).

## 4. Discussion

This study demonstrated that RA has a potent vasorelaxant effect. RA-induced vasorelaxation was determined to occur through the combined effects of endothelium-mediated and endothelium-independent mechanisms. RA-induced vasorelaxation was mediated by the PI3K/Akt/eNOS/NO/cGMP signaling pathway, various potassium channels (BK_Ca_, IK_Ca_, and Kv channels), calcium channels and membrane pores, and intracellular calcium stores.

Vasorelaxant activity is a crucial mechanism of action for antihypertensive agents. Naturally derived compounds with vasorelaxant activity have antihypertensive potential [30]. This study demonstrated the vasorelaxant activity of RA in the rat thoracic aorta. The maximum relaxation level was determined to be approximately 94%. After endothelial removal, the vasorelaxant effect decreased to approximately 47%. These data suggest that RA-induced vasorelaxation results from a combination of endothelium-dependent and endothelium-independent mechanisms.

It is well-known that endothelium-derived mediators are the most fundamental regulators of vascular tone [38,39]. Activation of the PI3K/Akt/eNOS pathway and stimulation of NO release from endothelial cells play an important role in regulating vascular function. NO, which diffuses into vascular smooth muscle cells, binds to the soluble guanylate cyclase (sGC) receptor, mediating vasorelaxation via cGMP and protein kinase G (PKG) [40,41]. Recent studies have reported that many vasoactive polyphenolic compounds derived from plants induce vasorelaxation in various vascular beds by activating the PI3K/Akt/eNOS/NO/cGMP pathway [42,43]. Having established that the endothelium is involved in RA-induced vasorelaxation, specific inhibitors were used to determine whether endothelium-derived mediators contribute to RA-induced vasorelaxation. Each inhibitor of the PI3K/Akt/eNOS/NO/cGMP/sGC/PKG pathway significantly blocked the vasorelaxant effect of RA. Another mediator derived from the endothelium that stimulates vasorelaxation is prostacyclin, also known as prostaglandin I2. Administration of indomethacin, a COX 1/2 inhibitor, did not significantly alter the vasorelaxant effect of RA. Zhou et al. reported that hydrogen peroxide–induced endothelial injury in rat thoracic aorta was ameliorated by RA, and this protective effect was mediated through AMPK and eNOS [44]. In the present study, however, AMPK inhibition did not modify the vasorelaxant action of RA. These findings suggest that the AMPK signaling pathway may contribute to the restoration of endothelial dysfunction under pathological conditions but does not participate in the vasorelaxant effect of RA in healthy vessels. Considering these data, it is concluded that endothelial integrity and endothelium-derived NO play a critical role in RA-induced vasorelaxation. Nevertheless, alterations in the underlying mechanisms may occur under pathological conditions.

Many common vascular diseases, such as hypertension and atherosclerosis, associated with increased vascular reactivity, are closely linked to potassium channels. It has also been suggested that endothelium-mediated mediators may play a role in the activation of potassium channels, thereby mediating vascular smooth muscle relaxation. Vascular smooth muscle contains numerous potassium channels of various subclasses. Activation of these channels mediates the vasorelaxant effects of many vasoactive substances and polyphenols. Activation of potassium channels increases the efflux of potassium, which is concentrated intracellularly. As a result of the hyperpolarization, voltage-gated calcium channels are inhibited, decreasing intracellular calcium levels and resulting in vascular smooth muscle relaxation [45,46,47,48,49,50]. In this study, the nonselective potassium channel blocker tetraethylammonium was first applied to thoracic aortic rings. Consequently, the RA-induced vasorelaxant effect was inhibited. The effects of selective inhibitors were then investigated. Incubations with the BK_Ca_ channel blocker iberiotoxin, the IK_Ca_ channel blocker TRAM-34, the nonselective voltage-gated potassium channel blocker 4-AP, and the Kv7.1–7.5 channel blocker XE-991 were performed separately. Each blocker significantly blocked RA-induced vasorelaxation. On the other hand, incubations with the SK_Ca_ channel blocker apamin, the inward-rectifying potassium channel blocker BaCl_2_, and the 2-pore potassium channel blocker anandamide did not produce a significant change in RA-induced vasorelaxant effect. These data clearly demonstrate that potassium channels, along with intact endothelium and NO, contribute significantly to the vasorelaxant effect of RA. Moreover, experiments performed in endothelium-denuded rings showed that tetraethylammonium, iberiotoxin, TRAM-34, 4-aminopyridine, and XE-991 administration significantly reduced RA-induced vasorelaxation. These data suggest that RA directly activates potassium channels. The contribution of potassium channel activation to vasorelaxation has been documented in previous studies. However, most earlier investigations focused on rosemary constituents other than rosmarinic acid. In terms of Kv channel activation, the results of the present study are consistent with previous findings. For example, Manville et al. [51] reported that a different rosemary (Salvia rosmarinus) extract, as well as carnosic acid and carnosol, induced relaxation in rat mesenteric artery preparations through the activation of Kv7 channels. In contrast, RA appears to activate additional potassium channel subtypes beyond Kv channels.

Calcium plays a critical role in vascular smooth muscle contraction. Therefore, it is expected that calcium also plays a role in the vasorelaxation mechanism. An increase in calcium in the cytoplasm triggers contraction, while a decrease in cytosolic calcium concentration mediates vasorelaxation. The increase in cytosolic calcium can be mediated by calcium influx through channels and membrane pores in the cell membrane, or it can be supported by intracellular sources such as the sarcoplasmic reticulum [52,53,54,55]. A series of experiments was conducted to demonstrate the role of these sources. First, we investigated whether RA incubation affected calcium-induced vasoconstriction. Calcium-induced contraction was significantly reduced after RA incubation. Similarly, vasoconstriction induced by the L-type calcium channel activator Bay K8644 was significantly blocked by RA incubation. The effect of nifedipine was also demonstrated as a positive control in these experiments. Additionally, the calcium ionophore ionomycin was used to assess whether RA affects calcium influx through membrane pores. Ionomycin-induced contraction was significantly inhibited by RA. Together, these data indicate that inhibition of extracellular calcium influx is critical for the vasorelaxant effect of RA.

Finally, the study investigated whether the PKC signaling pathway and the renin-angiotensin system are involved in the vascular tone-regulating effect of RA. The level of contraction achieved with the PKC activator was significantly reduced after RA incubation. Furthermore, RA incubation also significantly inhibited Ang II-induced vasoconstriction. This finding supports a previous study suggesting that RA inhibits the effects of Ang II. Prasannarong et al.’s study determined that RA prevented Ang II-induced increases in blood pressure and hyperglycemia [56]. Therefore, considering all these data, it was suggested that RA may exert antagonistic effects on the renin–angiotensin system under physiological and pathological conditions.

Although our study demonstrated that RA induces vasorelaxation via the PI3K/Akt/eNOS/NO/cGMP/K^+^ pathways, only functional experiments were performed, which represents a limitation. Therefore, the results should be interpreted with caution and considered preliminary. To strengthen these findings, future studies should confirm the effects of RA using complementary experimental approaches, such as genetic models or molecular techniques, including Western blotting, RT-PCR, or immunofluorescence, to directly assess pathway activation and protein expression.

In conclusion, the data obtained in this study demonstrated that rosmarinic acid has a potent vasorelaxant effect on the rat thoracic aorta. The primary mediators of the vasorelaxant effect induced by rosmarinic acid were determined to be the activation of intact endothelium, endothelium-derived nitric oxide (PI3K/Akt/eNOS/NO/cGMP signaling pathway), activation of potassium channels (BK_Ca_, IK_Ca_, and Kv channels), blockage of extracellular calcium influx (calcium channels and membrane pores), inhibition of calcium release from the sarcoplasmic reticulum (via SERCA and ryanodine receptors), inhibition of the protein kinase C signaling pathway, and inhibition of the effect of angiotensin II. It was concluded that rosmarinic acid, a natural polyphenolic compound of rosemary, holds promise for antihypertensive activity due to its potent vasorelaxant effect.

## Figures and Tables

**Table 1:** Drug concentrations used in the study and values of pD2 and Rmax on rosmarinic acid-induced vasorelaxation to different inhibitors.

Group	Effect	Conc.	PE Cont. (mg)	pD_2_	Rmax (%)	*n*	Ref.
Rosmarinic acid	Vasorelaxant effect	10^−10^–10^−5^ M	1362.63 ± 18.36	7.67 ± 0.04	93.70 ± 5.26	8	-
Rosmarinic acid (E-)	Vasorelaxant effect	10^−10^–10^−5^ M	1450.75 ± 45.44	5.26 ± 0.18	46.57 ± 7.88	8	-
LY294002	PI3K inhibitor	10 µM	1421.79 ± 38.56	4.17 ± 0.11	34.97 ± 4.47	8	[25]
Triciribine	Akt inhibitor	10 µM	1452.24 ± 38.54	4.57 ± 0.18	39.84 ± 5.93	8	[25]
L-NAME	eNOS inhibitor	100 µM	1452.53 ± 39.69	5.27 ± 0.14	47.81 ± 6.09	8	[36]
ODQ	sGC inhibitor	10 µM	1393.33 ± 15.42	5.02 ± 0.09	43.95 ± 7.74	8	[27]
Methylene blue	cGMP inhibitor	10 µM	1389.41 ± 14.72	5.57 ± 0.12	50.02 ± 7.75	8	[27]
KT5823	PKG inhibitor	1 µM	1419.00 ± 17.80	4.37 ± 0.11	37.60 ± 5.03	8	[29]
Dorsomorphin	AMPK inhibitor	1 µM	1425.27 ± 18.97	7.47 ± 0.16	88.57 ± 4.45	8	[33]
Indomethacin	Cyclooxygenase inhibitor	10 µM	1429.99 ± 18.01	7.53 ± 0.05	89.74 ± 5.13	8	[27]
SQ22536	Adenylate cyclase inhibitor	50 µM	1398.38 ± 34.35	7.59 ± 0.08	88.89 ± 3.98	8	[29]
Propranolol	Beta-adrenergic receptor blocker	1 µM	1435.93 ± 42.92	7.46 ± 0.05	88.07 ± 4.56	8	[27]
Atropine	Muscarinic receptor blocker	1 µM	1437.38 ± 36.42	7.65 ± 0.09	89.42 ± 4.49	8	[27]
Tetraethylammonium	Potassium channel blocker	1 mM	1372.99 ± 15,84	5.09 ± 0.15	45.46 ± 7.21	8	[27]
Iberiotoxin	BK_Ca_ channel blocker	10 nM	1442.11 ± 43.40	5.79 ± 0.12	56.13 ± 9.53	8	[34]
TRAM-34	IK_Ca_ channel blocker	1 µM	1436.32 ± 62.49	5.37 ± 0.14	48.89 ± 6.91	8	[35]
Apamin	SK_Ca_ channel blocker	1 µM	1431.24 ± 42.23	7.57 ± 0.05	88.68 ± 4.99	8	[35]
Glyburide	K_ATP_ channel blocker	10 µM	1422.27 ± 44.28	7.59 ± 0.06	86.79 ± 6.35	8	[27]
4-Aminopyridine	Kv channel blocker	1 mM	1416.24 ± 20.74	6.07 ± 0.11	59.99 ± 7.64	8	[27]
XE-991	Kv7.1–7.5 blocker	10 µM	1411.53 ± 69.88	6.37 ± 0.10	64.34 ± 5.54	8	[29]
Anandamide	K_2P_ channel blocker	10 µM	1429.44 ± 18.28	7.66 ± 0.06	86.98 ± 6.45	8	[37]
BaCl_2_	Kir channel blocker	10 µM	1438.80 ± 36.83	7.46 ± 0.07	87.82 ± 6.34	8	[27]

Noted: pD_2_: efficient concentration. Rmax: maximal relaxation. Results are expressed as mean ± SD (*n* = 8). Conc.: Concentration. E-: endothelium-denuded. PE cont.: Phenylephrine-induced contraction. Ref.: Reference.

## Data Availability

The raw data supporting the conclusions of this article will be made available by the authors upon request.
